# Adherence to the EAT-Lancet diet and change in cognitive functioning in older adults

**DOI:** 10.1007/s00394-025-03753-3

**Published:** 2025-08-12

**Authors:** Hanneke A. H. Wijnhoven, Marjolein Visser, Almar A. L. Kok, Margreet R. Olthof

**Affiliations:** 1 Department of Health Sciences, Faculty of Science, Amsterdam Public Health Research Institute, Vrije Universiteit Amsterdam, 1081 HV Amsterdam, The Netherlands; 2 Department of Epidemiology and Data Science, Amsterdam UMC location Vrije Universiteit Amsterdam, Amsterdam, The Netherlands; 3 Amsterdam Public Health Research Institute, Aging & Later Life Programme, Amsterdam, The Netherlands

**Keywords:** EAT-Lancet diet, Cognition, Older adults, Diet quality

## Abstract

**Purpose:**

Adherence to higher-quality diets has been linked to better cognitive function in older adults. Limited research exists on the EAT-Lancet diet, a healthy reference diet aligned with sustainability goals. This study examined the association between adherence to the EAT-Lancet diet and cognitive function and decline in older adults.

**Methods:**

Data from 1,371 participants aged 55–99 years from the Longitudinal Aging Study Amsterdam were analyzed. Adherence to the EAT-Lancet diet was assessed in 2014–2015 using a diet quality index based on a 238-item food frequency questionnaire. Cognitive domains—global cognition (MMSE), information processing speed (Coding task), episodic memory (15-Word Test), and executive function (Word Fluency)—were measured every three years (2011–2021) and converted to z-scores. Linear mixed models assessed associations between quintiles of adherence and cognitive function and decline (testing interaction with age), adjusting for demographic and lifestyle factors. Interaction by sex was tested.

**Results:**

Higher adherence to the EAT-Lancet diet was associated with better executive function (Q5 vs. Q1, β = 0.19 (0.07;0.30), P-trend 0.002), but not with episodic memory, information processing speed or global cognition. Higher adherence was associated with slower decline in information processing speed (Q5, Q3 vs. Q1, β = 0.01 (0.00;0.02), P trend 0.005), with no associations for other domains.

**Conclusion:**

Higher adherence to the EAT-Lancet diet is associated with better executive function and slower decline in information processing speed in Dutch older adults.

**Supplementary Information:**

The online version contains supplementary material available at 10.1007/s00394-025-03753-3.

## Introduction

The prevalence of dementia is projected to rise from 57.4 million cases in 2019 to 152.8 million by 2050 [1] posing a growing public health challenge. As effective treatments for dementia remain unavailable, research has increasingly focused on identifying modifiable risk factors [2]. While numerous lifestyle factors– such as smoking, alcohol consumption and physical inactivity - have been identified [2], the role of diet remains a topic of debate [2, 3]. Observational studies have shown that healthy dietary patterns such as the Mediterranean diet, the Dietary Approaches to Stop Hypertension (DASH) diet, and the Mediterranean-DASH Intervention for Neurodegenerative Delay (MIND) diet are associated with reduced cognitive decline and reduced dementia risk, though evidence from randomized controlled trials is limited [2–6].

Given its role in (cognitive) health, diet is increasingly viewed in a broader context, including its impact on sustainability - a consideration that is gaining importance given the growing interest in diets that support both individual and planetary health. Growing concerns about climate change and its impact on human health have increased the focus on sustainability in recent years [7]. International sustainability goals are closely related to the global food system, which has a major impact on the environment [8, 9]. To address this, in 2019 the international EAT-*Lancet* Commission has proposed the EAT-Lancet diet, a healthy reference diet al.igned with sustainability goals [10]. This diet emphasizes whole grains, fruits, vegetables, nuts, legumes, unsaturated oils, conservative amounts of seafood and poultry, and limits red meat, processed meat, added sugar, refined grains, and starchy vegetables.

While there is increasing evidence that the EAT-Lancet diet is associated with the a reduced risk of non-communicable diseases such as type 2 diabetes, cardiovascular disease and cancer [11, 12], the association with cognitive function remains under-researched. A recent review summarizing studies on individual components of the EAT-Lancet diet and cognitive function throughout life found that research in this area is still limited, with mixed findings and mostly weak associations [13]. However, initial studies have shown that higher adherence to the EAT-Lancet diet is associated with better cognitive function and less cognitive decline in older adults [14–16]. Therefore, this study aims to contribute to the evidence base by examining the longitudinal association between adherence to the EAT-Lancet diet and cognitive function and decline in Dutch older adults from the Longitudinal Aging Study Amsterdam (LASA). We hypothesize that higher adherence to the EAT-Lancet diet is associated with better cognitive function and slower cognitive decline. To test the robustness of the results and compare the strength of the associations, associations with the Mediterranean Diet Score (MDS) and Dutch Healthy Diet index 2015 (DHD15-index) were also examined as these diet indices were previously found to be associated with cognitive function and decline in a Dutch population [17].

## Methods

### Study design and participants

The data for this study were derived from the Longitudinal Aging Study Amsterdam (LASA), an ongoing prospective cohort study investigating the determinants, trajectories and consequences of physical, cognitive, emotional and social functioning in a representative sample of older adults from the general population of the Netherlands. Participants were randomly selected from municipalities across three culturally distinct regions. The study started in 1992/1993 with an initial cohort of adults aged 55–85 years. Since then, measurement cycles have been conducted approximately every three years. In 2002/2003 and 2012/2013, two additional cohorts of adults aged 55 to 65 years were incorporated into the original sample. Data collection involves a primary interview conducted by a trained interviewer, self-reported questionnaires, a medical interview, and clinical measurements. Detailed information on the study design and data collection procedures, including ancillary studies, is available in previous publications [18, 19]. Ethical approval for LASA and its ancillary studies was granted by the Medical Ethics Committee of the VU University Medical Center Amsterdam (METC number 2012/361), and all participants provided written informed consent.

For the present study, participants who completed a Food Frequency Questionnaire (FFQ) as part of the ancillary “LASA Nutrition and Food-Related Behavior Study” [20] were included. No additional inclusion or exclusion criteria were applied beyond the age range (55–85 years at initial inclusion).

**Fig. 1:**
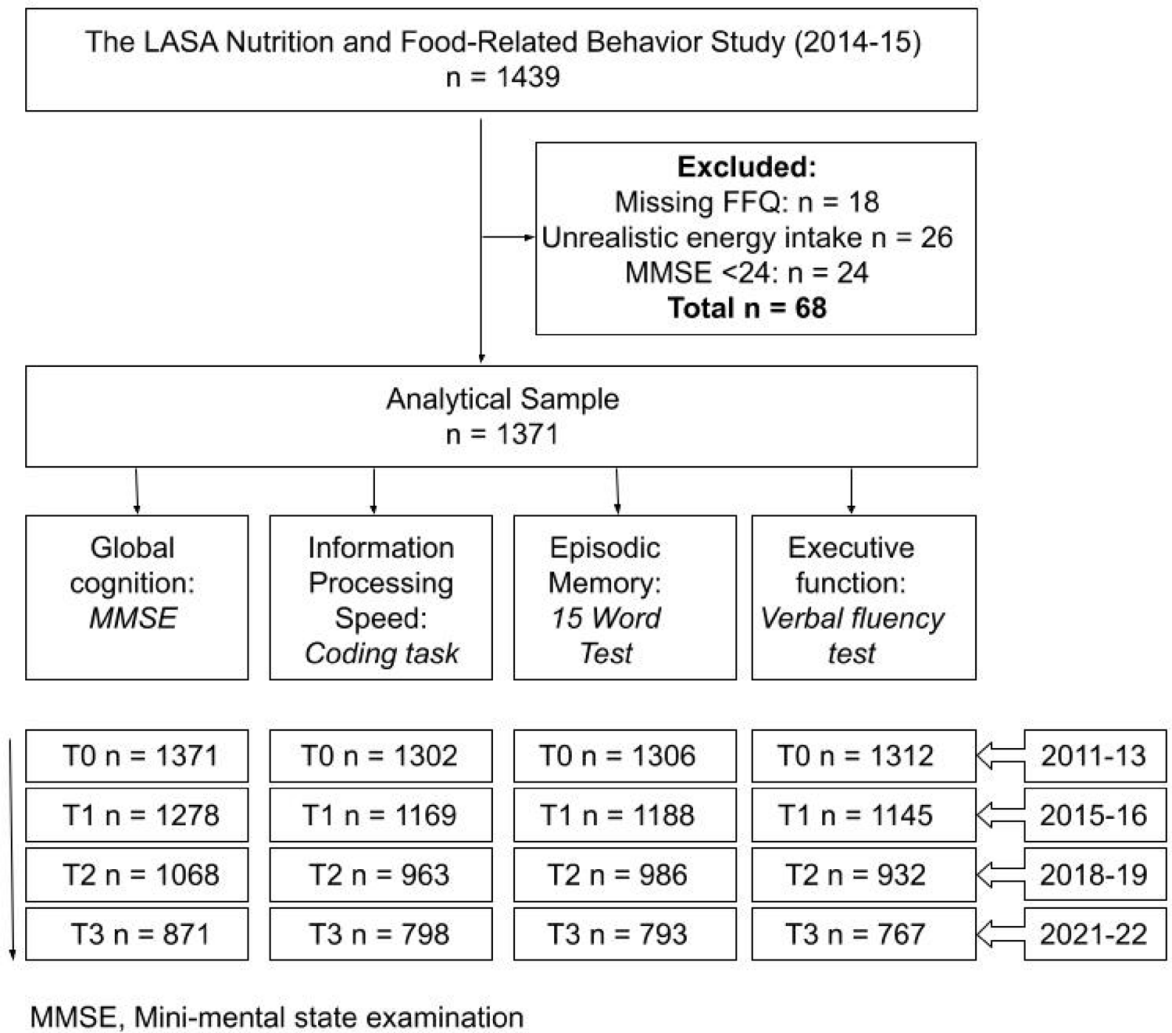
Flowchart with description of the study sample originating from the longitudinal aging study Amsterdam (LASA)

Figure 1 provides an overview of the study sample used in the present research. Dietary intake data were obtained during the ancillary “LASA Nutrition and Food-Related Behavior Study,” conducted in 2014/2015 between two regular LASA measurement waves [20]. In this ancillary study, a semi-quantitative FFQ was administered to 1,439 LASA participants. The 2011/2013 LASA measurement wave served as the baseline for cognitive assessments and covariates, with follow-up measurements conducted during the 2015/2016, 2018/2019, and 2021/2022 waves. Of the 1,439 participants who completed the FFQ, 18 were excluded due to having more than 10 missing responses on the FFQ. An additional 26 participants were excluded for reporting implausible energy intakes (< 500 kcal/day or > 3,500 kcal/day for women and < 800 kcal/day or > 4,000 kcal/day for men) [21]. To further ensure the validity of the FFQ, 24 participants were excluded due to potential cognitive impairment, as indicated by their Mini-Mental State Examination (MMSE) scores (< 24), which may have affected the reliability of their responses [22]. After these exclusions, the final study population consisted of 1,371 participants. Due to missing data on cognitive measures, the sample size varied slightly between analyses of different cognitive domains (Fig. 1).

### Measurements

#### EAT-Lancet diet index

Dietary intake was assessed using a 238-item semi-quantitative FFQ over a four-week period. This adapted FFQ, validated for a Dutch population, was developed for the Healthy Life in an Urban Setting (HELIUS) study [23]. The relative validity of the HELIUS FFQ was assessed in a subsample of 88 older adults (mean age 71.9 years) who also completed three 24-hour dietary recalls. For energy and macronutrients, group-level bias was ≤ 5%, with moderate to good Pearson’s correlations (*r* = 0.26–0.72) and moderate to high quintile agreement. Most micronutrients and food groups showed moderate validity (*r* = 0.3–0.5), with lower correlations for β-carotene, vitamin B1, fish, and grains [24]. Such levels of validity are typical for FFQs and are considered acceptable in dietary assessment research [25]. Daily intake of food items was calculated in grams per day by multiplying portion size by intake frequency. Adherence to the EAT-Lancet reference diet was measured using an index developed by Stubbendorf et al. in 2022 [26]. This index was based on intake levels and reference intervals of 14 food components defined in the EAT-Lancet diet (0–3 points per component; 0–42 points in total, higher scores indicating better adherence). These 14 food components were divided into two categories: emphasized intake (recommended foods) and limited intake (foods to minimize). We followed the approach as described in detail by Stubbendorf et al. [26]. Following the approach of Stubbendorff et al. [26], participants were categorized into five groups (quintiles) based on their index scores (≤ 17, 18–19, 20–21, 22–23, and ≥ 24). This classification was designed to obtain comparable group sizes.

#### Mediterranean diet score (MDS) and the Dutch healthy diet index 2015 (DHD-15)

To examine the robustness of the results - non-significant findings may also be due to e.g. insufficient variation in outcome measures, measurement errors, or lack of power– and compare the strength of the associations, secondary analyses were conducted with the MDS and the DHD15-index. The MDS was based on the methods outline by Panagiotakos et al. [27, 28]. The total score ranged from 0 (no adherence) to 55 (complete adherence). The DHD15-index, based on van Lee et al. [29], evaluated adherence to the Dutch dietary guidelines and includes 13 components (instead of 15) on a scale of 0–10 due to missing data on salt and type of coffee. The total score ranged from 0 (no adherence) to 130 (complete adherence). Both indices were categorized in quintiles (DHD15-index: ≤67, 68–78, 79–86, 87–95, and ≥ 96; MDS: ≤27, 28–31, 32–33, 34–36, and ≥ 37) following the same approach as the EAT-Lancet index.

#### Cognitive measurements

Cognitive function was assessed across several domains using standardized instruments, administered by trained interviewers during home visits. Global cognition was evaluated with the MMSE [30] while domain-specific cognitive functions were assessed through a Coding Task for processing speed [31], a 15 Words Test (15WT) for episodic memory [32, 33], and a Word Fluency Test for executive function [34]. Previous research has linked all these cognitive test outcomes with diet quality or eating behaviors [35–38].

The MMSE is a frequently used screening instrument for global cognitive impairment [30]. Scores range from 0 to 30 with a higher score indicating better performance.

Information processing speed was assessed using an adapted version of the Alphabet Coding Task-15, a letter substitution task [31]. Participants matched symbols to corresponding letters verbally during three one-minute trials. Unlike the original task, verbal responses were used instead of written ones to reduce delays caused by writing and to avoid difficulties in reading written responses during analysis [39]. The average score across trials, ranging from 2.0 to 49.3, was used to represent processing speed, with higher scores indicating faster processing speed.

Episodic memory was assessed using the 15 Word Test (15WT), a Dutch adaptation of the Rey Auditory-Verbal Learning Test [32, 33]. Participants were asked to recall as many words as possible from a list of 15 words over three trials and again after a 20-minute delay. Due to interview time constraints, the LASA study used three trials instead of the original five. Immediate recall scores (range 0–45), maximum recall (range 0–15), and delayed recall scores (range 0–15) were averaged for analysis (average scale range 0–25), with higher scores reflecting better memory. To minimize practice effects, two alternating word lists were used.

Executive function was measured through a verbal fluency test [34]. Participants were asked to generate as many words as possible starting with the letter “D” and to name as many animals as they could within one-minute intervals for each task. Repeated words were counted only once. Scores for the “D” words ranged from 0 to 36, while scores for animals ranged from 0 to 40. The average of the two scores (average scale range 0–38) was used as the measure of executive function, with higher values indicating better performance.

All cognitive scores were standardized into z-scores using the mean (M) and standard deviation (SD) from baseline measurements, enabling comparisons of the associations across domains. For the episodic memory test and executive function test with multiple components, z-scores were averaged.

#### Covariates

Information on age (years), sex at birth (male/female), education, partner status, body mass index (BMI), smoking status, alcohol use, number of chronic diseases and depressive symptoms, was collected through structured (medical) interviews during regular LASA measurement waves. We included covariates from all waves. Total energy intake (kcal/day) was calculated based on the data collected by the FFQ by linking each food item to one or more foods of a nutrient database based on the Dutch Food Composition Table (2011) [40]. Highest education completed with a degree was asked and was divided into: lower education (no completed education or primary general education); intermediate education (lower vocational education, secondary general education, intermediate vocational education or secondary general education); or higher education (academic education, higher vocational education or higher general education). Partner status was defined as ‘yes’ if participants had indicated that they lived with a partner. BMI was calculated by dividing body weight in kg by squared height in meters. Weight was measured to the nearest 0.1 kg on a calibrated scale and height to the nearest 0.001 meter with a stadiometer. Smoking status was categorized as never, former or current smoker. Alcohol consumption was assessed with a questionnaire developed by Statistics Netherlands [41] and defined as the number of alcoholic drinks per day, categorized into none, up to two, and more than two glasses. The number of glasses of alcohol per day was calculated by multiplying the frequency of alcohol consumption per week by the number of glasses each time, divided by seven. Physical activity over the past two weeks was assessed using the validated LASA Physical Activity Questionnaire [42]. Total physical activity was expressed in Metabolic Equivalent of Task (MET) hours per week, calculated by multiplying the frequency, duration, and MET scores of all reported activities. Number of chronic diseases was assessed by asking participants if they suffered from chronic non-specific lung disease, cardiac disease, peripheral arterial disease, diabetes mellitus, cerebrovascular accident, stroke, arthritis or cancer and categorized as none, 1 or 2, or > 2 chronic diseases. Lastly, depressive symptoms were measured using the self-report Center for Epidemiologic Studies Depression Scale (CES-D) [43].

#### Statistical analyses

Descriptive analyses were performed using SPSS Statistics (version 29, IBM Corp, Armonk, NY, USA). Baseline characteristics were presented for the total sample and stratified by quintiles of the EAT-Lancet index as means and standard deviations (SD) for normally distributed continuous variables, medians and interquartile ranges (IQR) for non-normally distributed variables, and percentages for categorical variables. In addition, the distribution (mean score and % of each scoring category) of the 14 food components of the EAT-Lancet index intake was presented for the total sample.

Linear mixed model analyses were conducted in Stata Statistical Software: Release 18 (StataCorp, 2021) to examine the association between quintiles of adherence to the EAT-Lancet diet, MDS and DHD15-index at baseline as categorical independent variables and the Z score of various cognitive measures assessed repeatedly over time as continuous dependent variables. Mixed model analyses accommodate missing data across time points, ensuring all available follow-up data are used. Normality of the residuals was evaluated using histograms and Q-Q plots, confirming normality in the regression residuals. All models employed an unstructured covariance matrix and included a random intercept at the participant level. A random slope for age (treated as a time-dependent variable) was incorporated when it improved model fit. Covariates were modeled as time-dependent variables, except for sex and education, which were treated as time-independent. All statistical tests were two-sided, with a significance level of *P* < 0.05.

Following the approach of Nooyens et al. [17], two types of models were used. The first model, referred to as the “level model,” assessed the association between quintiles of the diet score and cognitive function level. The second model, referred to as the “change model”, examined whether cognitive decline with age differed by quintiles of the diet score by including an interaction term between age and diet score (age × diet score). Both approaches were tested using two models: model 1 was adjusted for age, age², education and sex, while model 2 included additional adjustments for partner status, total energy intake, BMI, alcohol intake, physical activity, smoking status, number of chronic diseases, and depressive symptoms. A quadratic term for age (age²) was included to account for the previously observed non-linear relationship between cognitive function and age [17]. P-trend analyses were performed to evaluate the linear trend in cognitive level and decline across increasing quintiles of the EAT-Lancet index. To achieve this, a variable was created by assigning the median value of each quintile to all participants within that quintile. Mixed models were then used to assess associations, with this variable as the independent variable.

For all associations, potential effect modification by sex was first examined based on previous findings suggesting sex-dependent relationships between dietary pattern adherence and cognitive function in older adults [44]. This was done by including an interaction term in the fully adjusted model 2 (sex x diet score for the “level model” and sex x diet score x age for the “change model”). The three-way interaction was tested in the full model to ensure inclusion of all lower-order two-way interactions. Models were stratified by sex if there was statistically significant interaction (*P* < 0.05).

To illustrate differences in cognitive trajectories across quintiles of the EAT-Lancet index, predicted values from the “change model” (Model 2) were plotted as a function of age. Age and age² were centered at 55 years to facilitate interpretation. For clarity, the plot includes only the lowest and highest quintiles of the diet score, highlighting both cognitive function levels and age-related changes associated with the most extreme levels of dietary adherence.

Sensitivity analyses were conducted to assess the robustness of our findings using alternative analytical approaches. For the change model, we examined the interaction between EAT-Lancet quintiles and wave of assessment (1, 2, 3, or 4), adjusting for baseline age and baseline age². This approach allowed us to estimate the rate of cognitive decline per study wave, rather than per age year. For both the level and change model, we conducted an additional analysis in which participants with only a baseline cognitive measurement were excluded. This restriction allowed us to examine potential bias introduced by individuals with only a single baseline measurement in our main analysis.

## Results

Baseline characteristics of the total sample and stratified by quintiles of the EAT-Lancet index are presented in Table 1. The average age of participants was 67.3 ± 8.2 years, with 53% being female. Approximately one-third was higher educated. Mean BMI was 27.3 ± 4.3 kg/m², and mean energy intake was 2078 ± 575 kcal per day. Participants with the highest adherence to the EAT-Lancet diet were, on average, 1.7 years younger than those with the lowest adherence. They were also more often female (69% vs. 42%) and more often had a higher educational level (47% vs. 15%). Additionally, these participants smoked less frequently (8% vs. 17%), had a lower mean BMI (26.1 vs. 27.9 kg/m²), and a lower energy intake (1907 vs. 2288 kcal). They were also more physically active (median score 60.1 vs. 51.6 MET-hours per week). The average MMSE score at baseline was 28.4, indicating normal global cognitive function, with no difference between the lowest and highest adherence groups at baseline. Baseline scores for the coding task, 15-word test and verbal fluency test were higher in the highest compared to the lowest adherence group.

**Table 1:** Baseline sample characteristics of older adults from the longitudinal ageing study amsterdam, for the total sample and by quintiles of the EAT-Lancet index

		EAT-Lancet index (score 0–42):				
	Total sample	Lowest adherence ≤ 17	Low adherence 18–19	Medium adherence 20–21	High adherence 22–23	Highest adherence ≥ 24
n	1371	283	267	284	229	308
Age, y	67.3 ± 8.2	67.4 ± 8.3	68.2 ± 8.5	67.6 ± 8.2	67.6 ± 8.2	65.7 ± 7.5
Female sex, %	52.7	42.4	49.8	44.7	56.8	68.8
*Education, %*						
Lower	11.8	16.6	13.1	12.7	9.2	7.5
Intermediate	58.4	68.6	67.8	54.9	56.8	45.5
Higher	29.8	14.8	19.1	32.4	34.1	47.1
Living with partner, %	78.3	76.3	74.9	78.5	74.2	69.8
Body Mass Index, kg/m^2^	27.3 ± 4.2	27.9 ± 4.3	27.7 ± 4.3	27.6 ± 4.3	27.2 ± 4.4	26.1 ± 4.1
*Smoking status* ^1^ *, %*						
Current	11.7	16.8	17.1	7.3	9.5	7.9
Former	60.1	57.3	57.6	64.2	61.8	61.0
Never	28.1	25.8	25.3	28.5	28.6	31.2
*Alcohol glasses/week* ^1^ *, %*						
0	12.3	13.0	13.2	12.0	12.7	10.9
1–2	61.1	59.6	61.5	62.2	60.0	62.1
>2	26.6	27.4	25.3	26.8	27.3	27.0
MET-hours/week^1^	53.5 (46.4)	51.6 (50.0)	50.0 (45.6)	46.6 (50.5)	57.5 (47.9)	60.1 (42.3)
Energy intake, kcal/day	2078 ± 575	2288 ± 573	2084 ± 564	2084 ± 594	2034 ± 553	1907 ± 525
Depressive symptoms (CES-D score)	5 (8)	6 (7)	5 (9)	4 (8)	6 (8)	5 (6)
*Number of chronic diseases, %*						
0	30.7	29.7	30.3	30.3	31.0	32.1
1–2	39.5	42.8	36.7	38.0	35.4	43.5
>2	29.8	27.6	33.0	31.7	33.6	24.4
EAT-Lancet index	20.8 ± 4.2	15.3 ± 1.7	18.5 ± 0.5	20.5 ± 0.5	22.5 ± 0.5	26.6 ± 2.6
Mediterranean Diet Score	32.7 ± 4.8	29.4 ± 4.7	31.7 ± 4.2	32.6 ± 4.2	33.6 ± 4.4	35.8 ± 4.1
Dutch Healthy Diet index 2015	82.3 ± 16.1	70.3 ± 13.9	77.6 ± 13.5	81.6 ± 13.7	86.7 ± 13.2	95.0 ± 13.4
*Baseline cognitive function*:						
MMSE	28.4 ± 1.5	28.3 ± 1.5	28.3 ± 1.6	28.4 ± 1.4	28.5 ± 1.5	28.7 ± 1.3
Coding task^1^	29.4 ± 6.2	28.5 ± 6.4	28.7 ± 6.0	28.8 ± 6.1	29.6 ± 6.4	31.1 ± 6.09
15WT^1^	13.6 ± 3.6	12.8 ± 3.4	13.2 ± 3.6	13.3 ± 3.6	13.8 ± 3.5	14.6 ± 3.4
Verbal fluency^1^	16.9 ± 4.4	16.2 ± 4.3	16.5 ± 4.5	16.6 ± 4.2	17.2 ± 4.4	18.0 ± 4.7

Values are expressed as mean ± SD, median (IQR0) or percentages.

BMI, body mass index; CES-D, Center for Epidemiologic Studies Depression; IQR, interquartile range; MET, metabolic equivalent of task; MMSE, Mini-mental state examination; 15WT,15-word test.

^1^ Missing values ≤ 5% (no missing values for other variables).

**Table 2:** Mixed model analyses of cognitive level and cognitive decline by adherence to the EAT-Lancet diet with the study sample originating from the longitudinal aging study Amsterdam.^1^

		Adherence to EAT-Lancet index:	Model 1 β (95% CI)	Model 2 β (95% CI)
*Global cognition (MMSE)*				
n = 1371 model 1	Level^2^	Low	0.07 (-0.07, 0.21)	0.07 (-0.07, 0.21)
n = 1340 model 2		Medium	0.03 (-0.10, 0.17)	-0.01 (-0.14, 0.13)
		High	0.16 (-0.01, 0.31)	0.13 (-0.02, 0.27)
		Highest	0.07 (-0.06, 0.21)	0.04 (-0.10, 0.18)
		*P-trend*	*0.280*	*0.608*
	Change^2^	Low x age	-0.00 (-0.02, 0.01)	-0.01 (-0.02, 0.01)
		Medium x age	-0.01 (-0.02, 0.01)	-0.01 (-0.02, 0.01)
		High x age	0.01 (-0.01, 0.03)	0.01 (-0.01, 0.02)
		Highest x age	0.00 (-0.01, 0.02)	0.00 (-0.01, 0.02)
		*P-trend*	*0.530*	*0.462*
*Information Processing speed (Coding task)*				
n = 1339 model 1	Level^2^	Low	0.01 (-0.14, 0.15)	0.00 (-0.15, 0.14)
n = 1333 model 2		Medium	-0.03 (-0.18, 0.11)	-0.04 (-0.18, 0.11)
		High	0.06 (-0.10, 0.21)	0.04 (-0.12, 0.19)
		Highest	0.14 (-0.01, 0.29)	0.12 (-0.03, 0.27)
		*P-trend*	*0.051*	*0.101*
	Change^2^	Low x age	0.00 (-0.00, 0.02)	-0.00 (-0.01, 0.02)
		Medium x age	0.01 (0.00, 0.02)	**0.01 (0.00**,** 0.02)**
		High x age	0.01 (-0.00, 0.02)	0.01 (-0.00, 0.02)
		Highest x age	**0.02 (0.01**,** 0.03)**	**0.01 (0.00**,** 0.02)**
		*P-trend*	*0.004*	*0.005*
*Episodic memory (15-Word test)*				
n = 1341 model 1	Level^2^	Low	0.07 (-0.06, 0.20)	0.07 (-0.06, 0.20)
n = 1335 model 2		Medium	0.03 (-0.10, 0.16)	0.05 (-0.08, 0.18)
		High	0.12 (-0.02, 0.26)	0.10 (-0.04, 0.24)
		Highest	**0.13 (0.00**,** 0.27)**	0.13 (-0.01, 0.26)
		*P-trend*	*0.041*	*0.061*
	Change^2^	Low x age	-0.00 (-0.02, 0.01)	-0.00 (-0.02, 0.01)
		Medium x age	0.00 (-0.01, 0.02)	0.01 (-0.01, 0.02)
		High x age	0.01 (-0.00, 0.02)	0.01 (-0.00, 0.02)
		Highest x age	-0.01 (-0.02, 0.00)	-0.01 (0.02, 0.00)
		*P-trend*	*0.526*	*0.475*
*Executive function (Word fluency)*				
n = 1344 model 1	Level^2^	Low	0.08 (-0.04, 0.19)	0.10 (-0.02, 0.21)
n = 1338 model 2		Medium	0.02 (-0.09, 0.13)	0.05 (-0.07, 0.16)
		High	0.07 (-0.05, 0.19)	0.08 (-0.03, 0.20)
		Highest	**0.17 (0.06**,** 0.28)**	**0.19 (0.07**,** 0.30)**
		*P-trend*	*0.005*	*0.002*
	Change^2^	Low x age	0.00 (-0.01, 0.01)	0.00 (-0.01, 0.01)
		Medium x age	0.00 (-0.01, 0.01)	0.00 (-0.01, 0.01)
		High x age	0.00 (-0.01, 0.01)	0.00 (-0.01, 0.01)
		Highest x age	0.01 (-0.00, 0.01)	0.00 (0.00, 0.01)
		*P-trend*	*0.296*	*0.374*

^1^Standardized regression coefficients are calculated with lowest adherence to the EAT-Lancet diet as the reference group. Model 1 is adjusted for age, age^2^, education and sex; Model 2 is additionally adjusted for partner status, total energy intake, BMI, alcohol intake, physical activity, smoking status, number of chronic diseases, and depressive symptoms

^2^Positive coefficients denote better cognitive function (level model) or slower cognitive decline per age year (change model) compared with the cognitive function or cognitive decline in the lowest adherence group. Values printed in bold have a corresponding P value < 0.05

**Table 3:** Mixed model analyses of cognitive level and cognitive decline by adherence to the mediterranean diet score (MDS) and the Dutch healthy diet index 2015 (DHD15-index) with the study sample originating from the longitudinal aging study Amsterdam.^1^

		Adherence to diet index:	MDS β (95% CI)	DHD15-index β (95% CI)
*Global cognition (MMSE)*				
*n* = 1340	Level^2^	Low	0.06 (-0.07, 0.20)	**0.15 (0.01**,** 0.29)**
		Medium	-0.11 (-0.17, 0.14)	0.14 (-0.00, 0.28)
		High	0.04 (-0.11, 0.18)	0.10 (-0.05, 0.24)
		Highest	0.03 (-0.13, 0.20)	0.12 (-0.03, 0.27)
		*P-trend*	*0.941*	*0.156*
	Change^2^	Low x age	**-0.02 (-0.03**,** -0.00)**	-0.00 (-0.02, 0.01)
		Medium x age	-0.01 (-0.02, 0.01)	0.00 (-0.01, 0.02)
		High x age	0.00 (-0.01, 0.02)	-0.00 (-0.02, 0.01)
		Highest x age	0.01 (-0.03, 0.01)	0.00 (-0.01, 0.02)
		*P-trend*	*0.912*	*0.838*
*Information Processing speed (Coding task)*				
*n* = 1333	Level^2^	Low	0.08 (-0.06, 0.22)	0.04 (-0.11, 0.19)
		Medium	0.09 (-0.07, 0.24)	0.06 (-0.09, 0.21)
		High	0.13 (-0.02, 0.28)	0.01 (-0.14, 0.16)
		Highest	0.13 (-0.04, 0.29)	0.05 (-0.10, 0.21)
		*P-trend*	*0.098*	*0.659*
	Change^2^	Low x age	-0.00 (-0.01, 0.01)	-0.00 (-0.01, 0.01)
		Medium x age	0.01 (-0.01, 0.01)	-0.00 (-0.01, 0.01)
		High x age	0.01 (-0.00, 0.02)	-0.00 (-0.01, 0.01)
		Highest x age	0.00 (-0.01, 0.02)	0.00 (-0.01, 0.01)
		*P-trend*	*0.824*	*0.729*
*Episodic memory (15-Word test)*				
			*total sample*	*males*
*n* = 1335	Level^2^	Low	0.11 (-0.02, 0.23)	-0.00 (-0.16, 0.16)^4^
		Medium	0.03 (-0.11, 0.17)	**0.18 (0.03**,** 0.36)**
		High	**0.18 (0.04**,** 0.31)**	0.17 (-0.01, 0.35)
		Highest	**0.18 (0.03**,** 0.32)**	0.17 (-0.03, 0.37)
		*P-trend*	*0.013*	*0.020*
				*females*
		Low		**0.28 (0.07**,** 0.50)**^**4**^
		Medium		**0.23 (0.02**,** 0.44)**
		High		0.15 (-0.06, 0.36)
		Highest		**0.28 (0.07**,** 0.48)**
		*P-trend*		*0.086*
			*males*	*males*
	Change^2^	Low x age	-0.01 (-0.03, 0.00)	-0.01 (-0.02, 0.01)
		Medium x age	-0.01 (-0.03, 0.01)	-0.01 (-0.02, 0.01)
		High x age	0.01 (-0.03, 0.01)^3^	-0.00 (-0.02, 0.01)
		Highest x age	-0.01 (-0.03, 0.01)	-0.01 (-0.03, 0.01)^5^
		*P-trend*	*0.346*	*0.359*
			*females*	*females*
		Low x age	-0.00 (-0.02, 0.02)	0.01 (-0.01, 0.03)
		Medium x age	-0.01 (-0.02, 0.01)	0.02 (-0.00, 0.03)
		High x age	**0.02 (0.00**,** 0.04)**^**3**^	0.01 (-0.01, 0.03)
		Highest x age	0.01 (-0.01, 0.02)	**0.02 (0.00**,** 0.04)**^**5**^
		*P-trend*	*0.090*	0.033
*Executive function (Word fluency)*				
*n* = 1338	Level^2^	Low	0.04 (-0.07, 0.15)	0.10 (-0.02, 0.21)
		Medium	0.03 (-0.09, 0.15)	**0.12 (0.01**,** 0.24)**
		High	0.16 (0.04, 0.27)	0.11 (-0.01, 0.22)
		Highest	**0.20 (0.07**,** 0.33)**	**0.14 (0.02**,** 0.26)**
		*P-trend*	*0.000*	*0.028*
			*total sample*	*males*
	Change^2^	Low x age	-0.01 (-0.01, 0.00)	-0.01 (-0.02, 0.01)
		Medium x age	-0.00 (-0.01, 0.01)	-0.01 (-0.02, 0.01)^6^
		High x age	-0.00 (-0.01, 0.01)	-0.01 (-0.02, 0.00)
		Highest x age	-0.02 (-0.01, 0.01)	**-0.02 (-0.03**,** -0.00)**^**7**^
		*P-trend*	*0.418*	*0.018*
				*females*
		Low x age		0.01 (-0.00, 0.03)
		Medium x age		0.00 (-0.01, 0.01)^6^
		High x age		-0.00 (-0.02, 0.01)
		Highest x age		-0.01 (-0.01, 0.02)^7^
		*P-trend*		*0.776*

^1^Standardized regression coefficients are calculated with lowest adherence to the MDS or DHD15-index diet as the reference group. Associations are modelled stratified by sex in case of statistical significant interaction (*P* < 0.05). Models displayed are adjusted for age, age^2^, education, sex, partner status, total energy intake, BMI, alcohol intake, physical activity, smoking status, number of chronic diseases, and depressive symptoms

^2^Positive coefficients denote better cognitive function (level model) or slower cognitive decline per age year (change model) compared with the cognitive function or cognitive decline in the lowest adherence group. Values printed in bold have a corresponding P value < 0.05

^3^P-value sex interaction = 0.025. ^4^P-value sex interaction = 0.032. ^5^P-value sex interaction = 0.027

^6^P-value sex interaction = 0.044. ^7^P -value sex interaction = 0.010

**Fig. 2:**
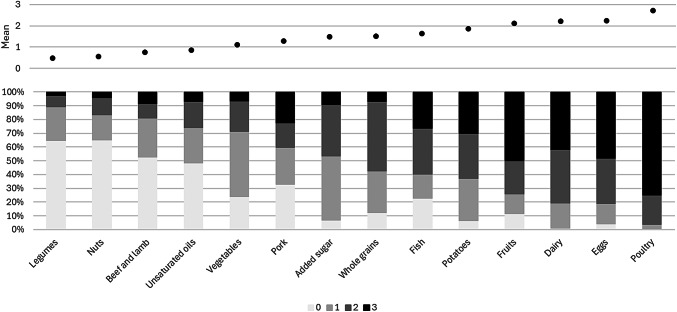
Distribution (mean score and % of each scoring category) of 14 food components of the EAT-Lancet index intake (0–3 points per component; higher scores indicating better adherence), with the study sample originating from the Longitudinal Aging Study Amsterdam (*n* = 1371)

Figure 2 shows the distribution of the scores of the 14 individual EAT-Lancet food components. Relatively higher adherence was found for poultry, eggs, dairy, and fruits, and lower adherence for legumes, nuts, beef and lamb, and unsaturated oils

**Fig. 3:**
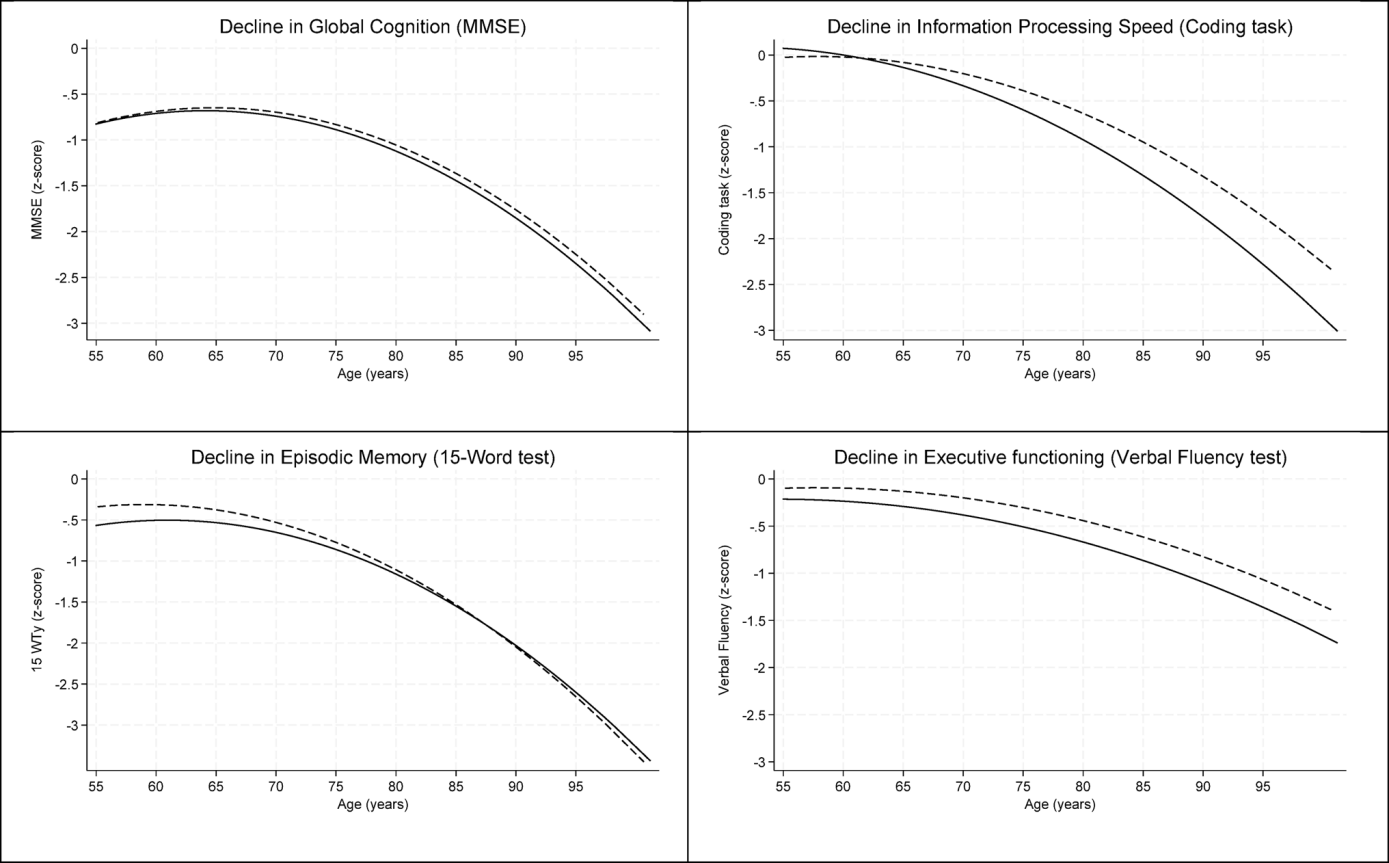
Cognitive decline with aging (plotted for age 55–100) in participants in the lowest (≤ 17) (*solid line*), and highest ≥ 24) (*dashed line*) quintile of adherence to the EAT-Lancet diet, with the study population originating from Longitudinal Aging Study Amsterdam and adjusted for age, age^2^, education, sex, partner status, total energy intake, BMI, alcohol intake, physical activity, smoking status, number of chronic diseases, and depressive symptoms

Table 2 provides an overview of the results of the “level” (without interaction quintiles x age) and “change” (with interaction quintiles x age) models for the association between adherence to the EAT-Lancet index and cognitive function and decline. No statistically significant interaction with sex was observed for any of the models. After adjustment for covariates, participants with the highest adherence to the EAT-Lancet diet (quintile 5) had an on average better executive function (β = 0.19, 95% CI: 0.07, 0.30) (P-trend = 0.002) compared to participants with the lowest adherence (quintile 1), while no differences were observed for episodic memory, information processing speed and global cognition. With respect to changes in cognitive decline by quintiles of the EAT-Lancet index, participants in the medium and highest groups exhibited a slower rate of decline per age year in information processing speed compared to those in the lowest adherence group (β = 0.01, 95% CI: 0.00, 0.02) (P-trend = 0.002), while no differences in decline per age year were observed for global cognition or the other cognitive domains. Figure 3 visualizes the modeled differences in cognitive function levels and age-related changes by the lowest and highest quintile of the EAT-Lancet diet score. Sensitivity analyses, which used study wave instead of age in the interaction term for the change models and excluded participants with only a baseline cognitive assessment in the level and change models, showed associations of similar strength compared to our main analyses (Supplementary Table 1).

Table 3 provides an overview of the results of the fully adjusted “level” (without interaction quintiles x age) and “change” (with interaction quintiles x age) models for the association between quintiles of adherence to the MDS and DHD15-index with cognitive function and decline. For some associations a statistically significant interaction with sex was found and models were subsequently stratified by sex. After adjustment for all covariates, participants with the highest adherence to the MDS or DHD15-ndex had an on average better episodic memory (MDS: β = 0.18, 95% CI: 0.03, 0.32; DHD15-index: β_males_ = 0.17, 95% CI: -0.03, 0.37 - β_females_ = 0.28, 95% CI: 0.07, 0.48) and executive function (MDS: β = 0.20, 95% CI: 0.07, 0.33; DHD15-index: β = 0.14, 95% CI: 0.02, 0.26) compared to participants with the lowest adherence, while no differences were observed for information processing speed and global cognition except for an association between low adherence to the DHD15-index and global cognition. With respect to changes in cognitive decline, female participants with the highest adherence to the MDS and DHD15-index exhibited a slower decline in episodic memory per age year compared to those with the lowest adherence (β = 0.02, 95% CI: 0.00, 0.04), while no differences in decline per age year were observed for males. No associations were observed for the other domains except for an opposite “negative” association between low compared to lowest adherence to the MDS and faster decline in MMSE and– in males only - high compared to lowest adherence to the DHD15-index and faster decline in executive function.

## Discussion

In this general population sample of older adults, higher adherence to the EAT-Lancet diet was associated with better executive function, as well as a slower rate of decline in information processing speed. No significant associations were observed with episodic memory and global cognitive function as measured by the MMSE. Comparable patterns were found for the MDS and the DHD15-index with some variation between domains; e.g., associations with (decline in) episodic memory instead of decline in information processing speed

Our findings align with previous research indicating that adherence to the EAT-Lancet diet is associated with better cognitive function and reduced cognitive decline in older adults [14–16]. However, the relationship between the EAT-Lancet diet and cognitive function differs across cognitive domains. Van Soest et al. [15] found higher adherence to the EAT-Lancet diet was associated with better global cognition (composite score) and executive function at baseline, and slower declines in global cognition, attention and working memory over 2 years among 630 cognitively healthy older adults, but no associations were found for episodic memory or information processing speed. Zhang et al. [14] reported that higher midlife adherence to the EAT-Lancet diet was associated with a reduced risk of poor global cognition based on the MMSE 20 years later in a cohort of 16,736 Chinese adults (mean age 54 y). This contrasts with our study, where the association with MMSE was in the same direction but not statistically significant, possibly due to our smaller sample size a shorter follow-up (9 years). Lastly, Goncalves et al. [16] observed - in a large cohort of 11,737 Brazilian adults (mean age 52 y, follow-up 8 years) - that higher adherence to the EAT-Lancet diet was associated with slower memory decline and slower global cognition (composite score) decline in high-income participants only, while no significant associations were found for executive function tested with verbal fluency tests and the trail-making test. Opposed to our study, they observed significant associations for the middle quintiles and not the highest quintile. Variations between studies may be explained by differences in cognitive tests, methods for calculating the EAT-Lancet diet index, characteristics of the study populations, follow-up time, and sample size.

We observed no consistent sex differences in the associations between diet quality and cognitive function. For the EAT-Lancet diet, associations were similar for males and females. In contrast, associations with episodic memory appeared somewhat stronger in females for both the MDS and DHD15-index. Moreover, only females with higher adherence to these dietary patterns showed a slower decline in episodic memory, while no such associations were observed in males. These findings differ from a previous study reporting no association between Western dietary pattern adherence and baseline cognitive function in females, but a negative association in males [44]. Some isolated findings—such as faster executive function decline in males with high DHD15 adherence - may reflect chance rather than systematic differences.

Our findings are also consistent with abundant evidence from previous observational studies showing that dietary patterns with a more plant-based focus like the Mediterranean, DASH, and MIND diets, which share similarities with the EAT-Lancet diet, are associated with a slower cognitive decline and lower risk of dementia [4–6]. Yet, results from randomized controlled trials have not consistently or robustly confirmed a potential impact, possibly due to limitations in study design [4–6]. When comparing the cognitive associations of the EAT-Lancet diet with those of the MDS and the DHD15-index in our study, results were generally comparable, with some variation observed across cognitive tests. Overall, our findings suggest that the cognitive benefits associated with the EAT-Lancet diet are similar to those of other diets that are more plant-based than regular Western diets.

Recognizing the complex interactions of nutrients and foods within dietary patterns, and the observation that dietary patterns show greater promise than individual nutrients and food groups to slow cognitive decline and reduce the risk of dementia [2–4], we did not evaluate individual nutrients or food components of the EAT-Lancet diet in relation to cognitive function. A previous review concluded that fruits and vegetables (beneficial), nuts (beneficial), and sugar (harmful), are particularly associated with cognitive function in older adults, but also that the evidence base is weak [13]. From a nutrient perspective, several nutrients may contribute to the cognitive aging benefits of the EAT-Lancet diet. Fruits and vegetables are rich in antioxidants and flavonoids, while fish, plant-based oils, and nuts provide polyunsaturated omega-3 fatty acids. These nutrients may reduce (brain) inflammation and oxidative stress, supporting brain health [45].

Our study, in line with previous research [14–16], suggests that the EAT-Lancet diet is not only associated with a reduced risk of non-communicable diseases such as type 2 diabetes, cardiovascular disease and cancer [11, 12] but also with better cognitive health in old age. However, besides cognitive health, it is also important to consider the impact of the EAT-Lancet diet on other health outcomes relevant for older adults. The limited intake of animal products in the EAT-Lancet diet raises concerns about potential deficiencies in nutrients abundant in animal-based foods, such as protein, vitamin B12, calcium, iron, and zinc [46, 47]. While the role of protein in brain health remains unclear [48], a lower intake of high-quality protein from animal sources may negatively impact physical function in older adults [49]. Also, a lower intake of calcium and vitamin B12 may negatively impact bone health [50, 51]. To fully optimize the benefits of the EAT-Lancet diet for physical health in older adults, it may be necessary to provide additional guidance to ensure adequate intake of nutrients. It is also worth noting that adherence to the EAT-Lancet recommendations was lowest for legumes in our sample, leaving room for a higher intake of plant-based protein with better adherence to this recommendation.

Strengths of this study include the longitudinal design with multiple repeated measures of cognitive outcomes and confounders, which enables the analysis of within-subject associations over time and enhances the study’s statistical power. Comparable results were obtained when baseline, instead of time-varying, confounders were modeled (data not shown). The use of a nationally representative sample of older adults enhances the generalizability of the findings. Further strengths are the use of a FFQ validated for the older Dutch population [24] and the inclusion of a comprehensive cognitive test battery that allowed us to examine associations across different cognitive domains. Additionally, the adjustment for a broad range of potential (time-varying) confounders reduced the risk of confounding bias and secondary analyses with the MDS and the DHD15-index allowed us to examine the robustness of the results. However, several limitations should be acknowledged. Although relevant confounding variables were adjusted for in the analyses, residual confounding may still exist and reverse causation cannot be excluded. Dietary intake was assessed only once at baseline, not capturing changes over time, which may have led to non-differential misclassification and an underestimation of associations. Compared to a previous Dutch study that performed similar analyses for the MDS and DHD15-index with a larger sample size and repeated dietary assessments [17], the lower power and greater measurement error in dietary intake in our study may explain the weaker associations observed in our study. Recall bias inherent in FFQs could also introduce inaccuracies, with participants possibly overreporting healthier habits, leading to differential misclassification and further underestimation of associations [52]. Although a standardized coefficient of 0.19 (0.07–0.30) for executive function represents a small effect, likely underestimated due to non-differential misclassification, even modest cognitive improvements in older adults can have significant long-term benefits, as also highlighted in the FINGER trial, where a small effect size (Cohen’s d 0.13 after 2 years) was interpreted as relevant in a public health context given its potential to delay cognitive decline and dementia incidence [53]. Lastly, while most associations point in the same direction, the large number of statistical comparisons still increases the risk of Type I errors, and results should therefore be interpreted with caution.

In conclusion, this study found that higher adherence to the EAT-Lancet reference diet was associated with better executive function and slower decline in information processing speed in Dutch older adults. These findings suggest that the EAT-Lancet diet is not only associated with a reduced risk of non-communicable diseases such as type 2 diabetes and cardiovascular disease but also with better cognitive health in old age.

## Supplementary Information

Below is the link to the electronic supplementary material.

Supplementary file1 (DOCX 448 kb)
