# Correction: Adherence to the EAT-Lancet diet and change in cognitive functioning in older adults

**DOI:** 10.1007/s00394-025-03804-9

**Published:** 2025-10-15

**Authors:** Hanneke A. H. Wijnhoven, Marjolein Visser, Almar A. L. Kok, Margreet R. Olthof

**Affiliations:** 1 Department of Health Sciences, Faculty of Science, Amsterdam Public Health Research Institute, Vrije Universiteit Amsterdam, 1081 HV Amsterdam, The Netherlands; 2 Department of Epidemiology and Data Science, Amsterdam UMC location Vrije Universiteit Amsterdam, Amsterdam, The Netherlands; 3 Amsterdam Public Health Research Institute, Aging & Later Life Programme, Amsterdam, The Netherlands

**Correction to: European Journal of Nutrition (2025) 64:252** 10.1007/s00394-025-03753-3

In the original version of this article, figure 2 was not correctly displayed. There should be 14 food items in the Figure 2. The correct figure 2 should have appeared as shown below

**Fig. 2:**
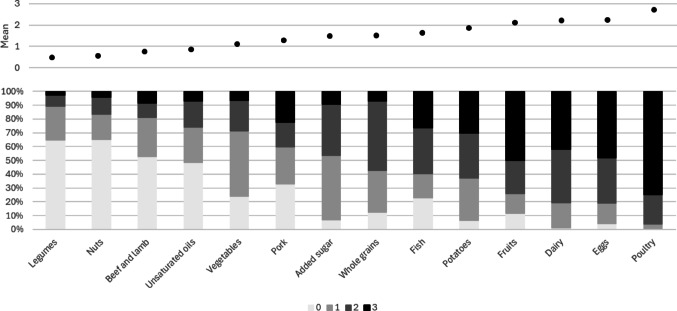
Distribution (mean score and % of each scoring category) of 14 foodcomponents of the EAT-Lancet index intake (0-3 points per component; higherscores indicating better adherence), with the study sample originating from theLongitudinal Aging Study Amsterdam (n=1371)

The original article has been corrected.

