# Supplementary material for: Adherence to the EAT-Lancet diet and change in cognitive functioning in older adults
Source: Eur J Nutr. 2025 Aug 12;64(6):252. doi: 10.1007/s00394-025-03753-3 (PMC12343652; doi:10.1007/s00394-025-03753-3)
Supplement: Supplementary file 1 — Supplementary file1 (DOCX 448 kb) [file 394_2025_3753_MOESM1_ESM.docx]

**Supplementary Table 1** Sensitivity analyses for Mixed model analyses of cognitive level and cognitive decline by adherence to the EAT-Lancet diet with the study sample originating from the Longitudinal Aging Study Amsterdam.^1^

|  |  | **Adherence to**  **EAT-Lancet index:** | **Model 3**  **β (95% CI)** | **Model 4**  **β (95% CI)** |
| --- | --- | --- | --- | --- |
| **Global cognition (MMSE)** | | |  |  |
| **n = 1340 model 3** | Level^2^ | Low |  | 0.08 (-0.06, 0.22) |
| **n = 1264 model 4** |  | Medium |  | 0.01 (-0.13, 0.15) |
|  |  | High |  | 0.14 (-0.01, 0.29) |
|  |  | Highest |  | 0.06 (-0.08, 0.20) |
|  |  | *P-trend* |  | *0.425* |
|  | Change^2^ | Low x time | -0.04 (-0.12, 0.04) | -0.01 (-0.02, 0.01) |
|  |  | Medium x wave | -0.03 (-0.11, 0.05) | -0.01 (-0.02, 0.01) |
|  |  | High x wave | 0.07 (-0.01, 0.16) | 0.01 (-0.01, 0.02) |
|  |  | Highest x wave | 0.02 (-0.06, 0.10) | 0.00 (-0.01, 0.02) |
|  |  | *P-trend* | *0.284* | *0.614* |
| **Information Processing speed (Coding task)** | | | |  |
| **n = 1333 model 3** | Level^2^ | Low |  | 0.02 (-0.13, 0.17) |
| **n = 1193 model 4** |  | Medium |  | -0.02 (-0.16, 0.13) |
|  |  | High |  | 0.04 (-0.12, 0.20) |
|  |  | Highest |  | 0.13 (-0.03, 0.28) |
|  |  | *P-trend* |  | *0.099* |
|  | Change^2^ | Low x age | 0.02 (-0.03, 0.06) | 0.01 (-0.00, 0.02) |
|  |  | Medium x wave | **0.05 (0.01, 0.09)** | **0.01 (0.00, 0.02)** |
|  |  | High x wave | 0.03 (-0.01, 0.07) | 0.01 (-0.00, 0.02) |
|  |  | Highest x wave | **0.07 (0.03, 0.10)** | **0.01 (0.00, 0.02)** |
|  |  | *P-trend* | *0.001* | *0.014* |
| **Episodic memory (15-Word test)** | | |  |  |
| **n = 1335 model 3** | Level^2^ | Low |  | 0.07 (-0.06, 0.21) |
| **n = 1198 model 4** |  | Medium |  | 0.05 (-0.09, 0.18) |
|  |  | High |  | 0.13 (-0.02, 0.27) |
|  |  | Highest |  | 0.11 (-0.03, 0.25) |
|  |  | *P-trend* |  | *0.130* |
|  | Change^2^ | Low x age | -0.01 (-0.06, 0.05) | -0.00 (-0.02, 0.01) |
|  |  | Medium x wave | -0.00 (-0.05, 0.06) | 0.00 (-0.01, 0.02) |
|  |  | High x wave | 0.02 (-0.04, 0.08) | 0.01 (-0.01, 0.02) |
|  |  | Highest x wave | 0.01 (-0.04, 0.06) | -0.01 (-0.02, 0.00) |
|  |  | *P-trend* | *0.563* | *0.270* |
| **Executive function (Word fluency)** | | |  |  |
| **n = 1338 model 3** | Level^2^ | Low |  | 0.10 (-0.02, 0.22) |
| **n = 1206 model 4** |  | Medium |  | 0.04 (-0.07, 0.16) |
|  |  | High |  | 0.08 (-0.04, 0.21) |
|  |  | Highest |  | **0.18 (0.05, 0.30)** |
|  |  | *P-trend* |  | *0.010* |
|  | Change^2^ | Low x wave | 0.01 (-0.03, 0.05) | 0.00 (-0.01, 0.01) |
|  |  | Medium x wave | -0.01 (-0.05, 0.03) | 0.00 (-0.01, 0.01) |
|  |  | High x wave | -0.01 (-0.05, 0.03) | 0.00 (-0.01, 0.01) |
|  |  | Highest x wave | 0.04 (-0.00, 0.07) | 0.00 (-0.01, 0.01) |
|  |  | *P-trend* | *0.097* | *0.514* |

^1^ Standardized regression coefficients are calculated with lowest adherence to the EAT-Lancet diet as the reference group. Model 3 includes the interaction term EAT-Lancet quintiles x wave of assessment (1, 2, 3, or 4), while adjusting for baseline age and baseline age^2^. Model 4 *excludes* participants with only a baseline wave assessment of cognition and adjusts for (time-varying) age and age^2^. Both models are additionally adjusted for education, sex, partner status, total energy intake, BMI, alcohol intake, physical activity, smoking status, number of chronic diseases, and depressive symptoms.

^2^ Positive coefficients denote better cognitive function (level model) or slower cognitive decline per study wave (change model 3) or age year (change model 4) compared with the cognitive function or cognitive decline in the lowest adherence group. Values printed in bold have a corresponding P value < 0.05.
